# Progress in Research on the Mechanism of GABA in Improving Sleep

**DOI:** 10.3390/foods14223856

**Published:** 2025-11-11

**Authors:** Shuyu Li, Yanhui Li, Chunxu Xue, Ying Zhang, Tong Tong, Zijun Ouyang, Dong Liu, Jun Cai, Haiyan Sun

**Affiliations:** 1 School of Life and Health Sciences, Hubei University of Technology, Wuhan 430068, China; lishuyu@mail.szpu.edu.cn; 2 School of Food and Drug, Shenzhen Polytechnic University, Shenzhen 518055, China; liyanhui1@szpu.edu.cn (Y.L.); xuechunxu@outlook.com (C.X.); zhangying20230198@szpu.edu.cn (Y.Z.); tongtong@szpu.edu.cn (T.T.); ouyangzijun@szpu.edu.cn (Z.O.); liudongsz@szpu.edu.cn (D.L.)

**Keywords:** GABA, probiotics, sleep regulation, gut–brain axis, blood–brain barrier (BBB), metabolic pathway, functional foods

## Abstract

Sleep disorders represent a growing global health concern with significant socio-economic impacts. GABA, a natural bioactive compound abundant in various fermented foods, especially probiotic-fermented foods, has garnered increasing attention for its potential to improve sleep quality. This review systematically elucidates the multi-pathway mechanisms by which GABA regulates sleep, focusing on (1) indirect modulation of central sleep–wake circuits via the gut–brain axis through vagal nerve, neuroendocrine, and immune pathways; (2) potential entry into the brain by leveraging the dynamic permeability of the blood–brain barrier (BBB) and transporter-mediated active transport; and (3) metabolic conversion into active substances like γ-hydroxybutyrate (GHB), which synergistically optimizes sleep architecture via multiple receptor systems and energy metabolism. Furthermore, we summarize the sleep-promoting effects of GABA-enriched foods observed in animal and clinical studies and discuss emerging applications, including high-GABA-yielding probiotics and personalized nutrition strategies for sleep intervention. This review provides a theoretical basis and innovative directions for the development of GABA-based functional foods and sleep health management.

## 1. Introduction

Sleep disorders, characterized by difficulties in falling asleep, maintaining sleep, and poor sleep quality, have become a prevalent worldwide issue [1]. Chronic sleep deprivation can lead to various ailments, including anxiety, depression, and metabolic syndrome, imposing a substantial socio-economic burden [2]. Current mainstream interventions, such as benzodiazepines, are associated with risks of dependency and cognitive side effects, highlighting the need for safe and effective alternatives. GABA, a naturally occurring non-protein amino acid widely found in grains, legumes, plants, and microorganisms, is increasingly being studied and applied for non-pharmaceutical sleep improvement.

GABA is the primary inhibitory neurotransmitter in the central nervous system (CNS) of humans and other mammals. By activating GABA type A (GABA_A_), type B (GABA_B_), and type C (GABA_C_) receptors, it mediates fast and slow synaptic inhibition, regulates neuronal excitability, and plays a key role in maintaining neural homeostasis, regulating sleep–wake cycles, and managing mood [3]. The sleep-improving effects of dietary GABA or GABA-rich probiotics are being increasingly validated in both human and animal models [4]. For instance, studies indicate that GABA supplementation significantly shortens sleep latency and extends non-rapid eye movement (NREM) sleep in mice. Evidence from human studies shows that certain probiotics, including *Lactiplantibacillus plantarum* TWK10, improve sleep quality via mechanisms involving increased gut GABA levels and gut–brain axis regulation [5].

The mechanisms underlying GABA’s sleep-improving effects are multifaceted. While traditional research has focused on the direct modulation of neurotransmitter systems (e.g., enhancing GABA_A_ receptor-mediated Cl^−^ influx, promoting neuronal hyperpolarization), recent studies propose additional pathways for modulation. These include indirect influences on sleep quality via gut–brain axis signaling, transport across the blood–brain barrier (BBB), and regulation by metabolites like short-chain fatty acids (SCFAs) [6,7]. However, a unified conclusion and systematic summary are still lacking. Therefore, this review synthesizes recent findings to systematically elaborate the mechanisms of GABA in sleep improvement from three perspectives: the gut–brain axis, the blood–brain barrier, and metabolic pathways. In parallel, the development of GABA-enriched functional foods (e.g., probiotic-fermented foods) has emerged as a promising strategy for sleep health management. These products leverage various food matrices and processing technologies to enhance GABA concentration, bioavailability, and efficacy. It also summarizes the roles of different food matrices and processing technologies in enhancing GABA concentration, activity, and delivery efficiency, aiming to provide a theoretical reference and practical insights for developing sleep-improving functional foods and targeted nutritional interventions. It is important to note that although the oral bioavailability of GABA and its ability to cross the blood–brain barrier remain subjects of debate, clinical studies employing typical dietary doses (generally in the range of 100–300 mg/day) have consistently reported significant improvements in sleep outcomes [8]. This suggests that its efficacy may not rely solely on direct brain entry, but is rather closely associated with indirect mechanisms-namely, the gut–brain axis, dynamic BBB regulation, and metabolic pathways, as will be elaborated in subsequent sections. By exploring these multi-dimensional mechanisms in conjunction with evidence from studies employing these typical doses, this review aims to bridge the gap between mechanistic theory and practical food applications [9].

## 2. Mechanisms of GABA in Sleep Improvement via the Gut–Brain Axis

Early research primarily focused on GABA’s neurotransmitter function, emphasizing its direct inhibitory effects on the CNS and its role in promoting sleep through the GABAergic pathway [10,11]. Recent interdisciplinary studies combining food science and neuroscience have revealed that GABA can indirectly influence central sleep regulation through the gut–brain axis. The gut–brain axis is a complex, bidirectional communication system between the gastrointestinal tract and the brain, mediated by neural pathways (e.g., the vagus nerve), neuroendocrine pathways, and immune signals [12]. Dietary GABA or GABA-enriched probiotics can exert regulatory roles within this system, facilitating cross-barrier communication from the gut to central sleep-regulating centers.

### 2.1. Vagus Nerve Pathway

The vagus nerve is a crucial direct neural pathway connecting the gut and the brain [13,14]. Initial debate existed regarding whether orally administered exogenous GABA could improve sleep, considering its exposure to gastric acid and intestinal bile before reaching the gut, and questions about sufficient bioavailability and BBB penetration [15]. However, research has shifted towards understanding how GABA, especially when produced locally in the gut, can influence the brain via the vagus nerve, circumventing the BBB issue. The influence of GABA on sleep via the vagus nerve involves both direct and indirect mechanisms. The most direct pathway is through the binding of these gut-derived GABA molecules or their precursors to GABA receptors (e.g., GABA_A_ or GABA_B_) on vagal nerve endings [16,17]. Activated signals then travel via afferent vagal fibers to the brainstem, particularly the nucleus tractus solitarius (NTS), and are relayed to the cerebral cortex and sleep-related brain regions like the thalamus, hypothalamus, and brainstem reticular formation [18]. Such vagal signaling mechanisms circumvent the constraints imposed by the BBB, allowing gut-derived GABA to influence CNS function effectively [19]. In addition to direct receptor activation, GABA and GABA-producing probiotics may exert influence on vagal function through a variety of indirect pathways, thereby modulating gut–brain communication [20].

These indirect mechanisms operate in a layered manner to ultimately alter vagal afferent signaling. First, the modulation of gut microbiota represents another crucial indirect pathway. Probiotics, including specific strains like *Lbp. plantarum*, can reshape the gut microbial community, thereby enhancing GABA production and sustaining a gut environment conducive to healthy vagal nerve activity [21,22]. A well-functioning intestinal barrier, fortified by probiotics that strengthen epithelial tight junctions, reduces intestinal permeability and systemic inflammation, which in turn sustains vagal nerve activity [23]. In contrast, gut dysbiosis disrupts this balance, triggering an inflammatory response that can interfere with vagal function; interventions aimed at rebalancing the gut microbiota can alleviate inflammation and indirectly restore vagal tone [24]. Second, microbial metabolites serve as key intermediaries. Short-chain fatty acids (SCFAs)—such as butyrate, acetate, and propionate, which are derived from microbial fermentation of dietary fiber-modulate intestinal inflammation and immune responses, thereby indirectly influencing vagal afferent signaling [25]. The collective action of these indirect pathways means that modulating the gut microbiota can directly influence the activity of intrinsic sensory neurons within the gut wall, thereby altering the signals transmitted to the central nervous system via the vagus nerve [26]. Recent studies show that specific GABA-producing probiotics from fermented dairy, grains, and plant-based beverages can significantly increase GABA content during fermentation [19]. The GABA produced by these probiotics is considered a potential “postbiotic” that can function via the gut–brain axis [21]. For example, LB-GABA, derived from fermented soy milk, was shown to extend sleep duration by 8–9% in fruit flies and 59% in mice, while reducing their nocturnal activity levels [27]. Supporting the role of this gut-vagus-brain axis, certain probiotics (e.g., *Lacticaseibacillus rhamnosus*) can enhance intestinal GABA synthesis, which then activates GABAergic neurons in the NTS via the vagus nerve, inhibiting the activity of wake-promoting hypothalamic nuclei (e.g., tuberomammillary nucleus (TMN)), thereby promoting NREM sleep [6]. Notably, vagotomy in infected mice effectively controlled neurotransmitter disturbances, increased colonic GABA content, and enhanced the abundance of GABA-producing *Bacteroides* and *lactobacilli*, underscoring the vagus nerve’s role in maintaining GABA-related regulatory mechanisms [13]. These findings suggest that GABA can influence central GABAergic neuron function through the gut–brain axis and vagal activity, thereby improving sleep [11,28]. Despite the accumulating evidence from preclinical models, several limitations persist in the current body of research regarding the vagal pathway. First, the majority of existing evidence is derived from preclinical studies, including animal models and in vitro experiments, whereas high-quality human clinical data remain scarce and insufficient to establish definitive conclusions. Second, the specific molecular mechanisms underlying GABA signal transmission through the vagus nerve, particularly the key signaling molecules, regulatory pathways, and synaptic modulation processes involved, have not been fully elucidated, leaving critical gaps in our understanding of this neurochemical communication axis. Third, the influence of individual variability, such as interindividual differences in gut microbiota composition, genetic backgrounds, and baseline neurophysiological states, on the efficacy and dynamics of vagus nerve-mediated GABA signaling remains largely uncharacterized and thus requires systematic investigation.

To address these limitations, future research should employ integrative approaches-such as combining multi-omics with neuroimaging-to holistically map the molecular-to-systemic networks of vagus nerve-mediated GABA signaling in sleep regulation. 

### 2.2. Neuroendocrine Pathway

GABA may also improve sleep through the neuroendocrine system, particularly the hypothalamic–pituitary–adrenal (HPA) axis, which regulates hormone secretion and stress responses [29]. Chronic stress can overactivate the HPA axis, resulting in elevated cortisol levels that disrupt sleep [30]. Exogenous GABA or GABA produced by gut microbes may indirectly regulate HPA axis function to improve sleep quality.

GABA might act on receptors on enteroendocrine cells (EECs), initiating intracellular signaling that indirectly affects the synthesis and release of neuroendocrine hormones, thereby modulating neuronal activity in sleep–wake-related brain regions. EECs secrete various gut peptides like Ghrelin, Cholecystokinin (CCK), Glucagon-like peptide-1 (GLP-1), and Neuropeptide Y (NPY), which regulate appetite, energy metabolism, stress response, and interact complexly with the HPA axis [31,32]. GABA may modulate the release of these peptides, consequently influencing HPA axis activity. For instance, Ghrelin is a promising therapeutic target for depression, which often co-occurs with HPA axis dysfunction and sleep issues, indirectly supporting this potential mechanism [32,33].

The neuroendocrine mechanism is complex, involving the gut microbiota, GABAergic signaling, and intricate the regulation of the HPA axis. Studies also show that GABA-rich fermented milk can modulate gut microbiota and increase SCFA levels, thereby indirectly suppressing HPA axis overactivity [34]. Thus, GABA’s effects on neuroendocrinology are multidimensional and must be considered comprehensively in sleep mechanism research.

### 2.3. Immune Pathway

Maintaining intestinal immune homeostasis is fundamental for GABA’s sleep effects via the gut–brain axis. GABA or specific GABA-producing probiotics can indirectly affect sleep by modulating immune status and gut barrier function [35]. Gut dysbiosis can lead to the release of pro-inflammatory cytokines (e.g., interleukin-1β (IL-1β), tumor necrosis factor-α (TNF-α), interleukin-6 (IL-6)), which can enter the brain via the vagus nerve or circulation, inhibit GABAergic neuron activity, and disrupt sleep [36]. Supplementing with GABA or certain probiotics (e.g., *lactobacillus* and *Bifidobacterium*) can increase anti-inflammatory cytokines (e.g., IL-10), reduce levels of pro-inflammatory factors in the gut and CNS, help restore gut barrier integrity, and reduce translocation of lipopolysaccharide (LPS) into the bloodstream due to barrier damage. As an “endotoxin,” LPS is a key molecule that induces chronic inflammation. Reducing systemic LPS levels can lower central “micro-inflammation,” decrease the upward regulation of neural excitation by inflammatory factors [37], indirectly enhance GABA’s inhibition of “wake-related nuclei,” reduce neural excitability, facilitate the transition from wakefulness to sleep, and ultimately alleviate insomnia symptoms [38,39]. Recent studies provide specific details: consuming of GABA-enriched fermented dairy (15 mg/100 g) for 4 weeks significantly reduced serum IL-6 by 28.5%, increased IL-10 by 37.2%, and shortened sleep latency by 22% [40]. Daily intake of GABA-rich green tea (50 mg/200 mL) reduced serum LPS-binding protein (a gut permeability marker) by 19%, plasma TNF-α by 32%, and increased polysomnography-measured slow-wave sleep duration by 18 min [21]. GABA-enriched rice bran inhibited the TLR4-MAPK/NF-κB pathway, reducing TNF-α and IL-6 expression in LPS-induced RAW264.7 macrophages [41]. Fermented brown rice with *Aspergillus oryzae* (FBRA) alleviated inflammation in a TPA-induced mouse ear edema model, reducing the expression of COX-2 and iNOS [42]. The core mechanism involves anti-inflammatory actions and gut barrier repair, indirectly strengthening GABA’s inhibition of neural excitation. However, most studies focus on specific matrices or strains, lacking data on population diversity, and long-term safety, which warrants further research for the precise functional food development.

**Figure 1:**
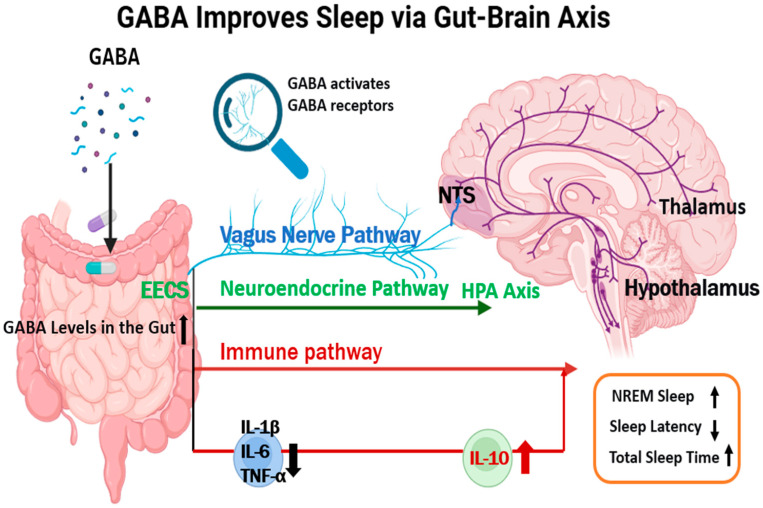
GABA Improves Sleep via the Gut–Brain Axis. Vagus nerve pathway-pathway of vagus nerve; Neuroendocrine pathway-neuroendocrine pathway; Cerebral cortex-cerebral cortex; Thalamus-thalamus; Hypothalamus-hypothalamus; HPA-Axis-hypothalamic–pituitary–adrenal axis; Gut-gut; Immune pathway-immune pathway; IL-10-interleukin-10. All the black solid arrows in the figure represent the regulatory and transmission pathways of GABA in improving sleep via the gut—brain axis, the dashed arrows indicate the influence pathway of GABA on relevant brain structures, and the arrows related to the HPA Axis and IL-10 represent the interaction direction in the sleep improvement process.

Current evidence from in vitro, animal, and some human studies supports GABA’s involvement in sleep regulation via three core gut–brain axis pathways: vagal nerve signaling that activates central sleep regions; HPA axis regulation, which mitigates stress-induced sleep interference; and improved gut immune homeostasis, which reduces inflammatory impacts on neural excitation. Based on these, we summarize the mechanism in Figure 1. 

## 3. Mechanisms of GABA Penetration Through the Blood–Brain Barrier

The traditional view holds that GABA rarely crosses the BBB to modulate central GABAergic systems directly. However, recent studies reveal dynamic regulatory mechanisms of BBB permeability, suggesting the potential for GABA transport.

### 3.1. Dynamic Regulation of BBB Permeability

The BBB is a crucial structure that protects the CNS, selectively regulating substance passage to maintain microenvironmental homeostasis [43]. Comprising brain microvascular endothelial cells (BMECs), pericytes, and astrocytes, which are connected by tight junctions, it restricts the paracellular passage of hydrophilic molecules [44]. Efflux transporters like P-glycoprotein (P-gp) further limit brain entry of substances [45]. This selectivity led to the perception of the BBB as a static, nearly impermeable barrier, suggesting that exogenous GABA must be synthesized intracerebrally from precursors, such as glutamate, to act effectively [46].

Advances in molecular biology challenge this view. BBB integrity is dynamically regulated, closely associated with permeability to substances like GABA. The expression and integrity of tight junction proteins (e.g., Claudin-1, Claudin-5, Occludin) are key. For instance, endothelial neurogranin (Ng) deficiency in mice significantly reduced these proteins, thereby impairing BBB integrity and increasing permeability [47]. Pathological conditions, such as ischemic stroke, neuroinflammation, and neurodegeneration, damage BBB integrity, increasing permeability and allowing usually restricted substances into the brain [48]. However, recent studies also show that in healthy individuals, the BBB’s permeability can be influenced by various factors, including diet and exercise. For example, certain dietary components, such as GABA, may enhance the expression of specific transporters (e.g., GATs) on BBB endothelial cells, thereby facilitating GABA transport into the brain [49]. Heterogeneous permeability increases are also observed in brain tumors [50], indicating that the BBB is dynamically regulated, rather than being absolutely closed. In summary, the recognition of the BBB’s dynamic nature represents a paradigm shift from the traditional view of its absolute impermeability. This revised understanding opens up the theoretical possibility that dietary interventions, such as specific nutrients or functional food components, could gently modulate BBB transport capacity even under non-pathological conditions. A key focus of future research will be to elucidate these physiological regulatory mechanisms, which is essential for developing safe and controllable functional food strategies that target the BBB pathway.

### 3.2. GABA Transport Pathways Across the BBB

Based on the recognition of the dynamic regulation of the BBB, researchers have begun to explore potential transport pathways for GABA across this barrier. Current evidence suggests that the trans-BBB transport of GABA involves several mechanisms. Active transport represents one of the key mechanisms. Studies indicate that GABA transporters (GATs) are likely present on BBB endothelial cells [43]. Research using the conditionally immortalized mouse brain capillary endothelial cell (TM-BBB) model has demonstrated saturable and inhibitable GABA uptake, suggesting a role for GATs (such as GAT2/BGT-1) in GABA transport at the BBB. In healthy individuals, Exogenous GABA (such as through dietary intake) may indirectly regulate GABA levels in the brain by influencing the expression and activity of GATs on the blood–brain barrier [51]. Furthermore, specific amino acid transporters may also facilitate GABA transport, suggesting the involvement of multiple solute carrier family members in its active uptake [52]. Regarding the paracellular pathway, GABA may passively diffuse into the brain parenchyma through the gaps between endothelial cells, particularly under conditions of impaired BBB integrity or increased permeability. For instance, the Wnt/β-catenin signaling pathway plays a crucial role in maintaining the integrity of the BBB and its normal function. Dysregulation of this pathway can increase barrier permeability, potentially facilitating the paracellular passage of substances like GABA [53]. Studies showing that γ-glutamylcysteine (γ-GC) ameliorates BBB permeability by modulating the Wnt/β-catenin pathway [54]. indirectly support the notion that modulating tight junctions could influence GABA’s paracellular transport. Additionally, specific receptors on BBB endothelial cells, such as the transferrin receptor, can initiate receptor-mediated transcytosis. By conjugating GABA to transferrin or its antibodies, this pathway could potentially be leveraged to enhance GABA concentration in the brain, offering an additional route for GABA or its complexes to enter the brain [55]. Several critical gaps persist in our understanding of GABA transport across the BBB. Foremost, the functional expression, distribution, and regulatory mechanisms of GABA transporters (e.g., GAT2/BGT-1) in the human BBB remain unconfirmed, lacking crucial validation from human-derived models such as iPSC-derived endothelial cells and direct in vivo evidence from techniques like PET imaging. Furthermore, it is unclear whether BBB permeability and associated GABA transporter activity undergo rhythmic fluctuations in concert with the sleep–wake cycle, a dynamic that could critically influence the temporal efficiency of GABA entry into the brain. Finally, a comprehensive mapping of the heterogeneity in transporter distribution and function across different brain regions-especially key sleep-regulating nuclei-is urgently needed and could be elucidated using advanced techniques like single-cell sequencing.

### 3.3. Innovative Delivery Technologies for Enhancing GABA Brain Permeability

Building on the understanding of GABA transport mechanisms across the BBB, researchers have recently pursued targeted innovations in delivery technologies to enhance the brain permeability of exogenous GABA and apply these strategies to treat neurological disorders such as sleep disturbances and anxiety.

Innovations in oral delivery systems provide a crucial direction for overcoming the limitations of GABA’s gastrointestinal stability and poor BBB penetration [56]. The application of nanotechnology is particularly noteworthy. By designing nanoparticles with specific surface properties or targeting ligands, it is possible to exploit the BBB’s natural transport mechanisms or induce temporary disruption to enhance GABA delivery to the brain [43]. Food-grade carriers, such as whey protein nanoparticles and chitosan microspheres, show significant potential for oral delivery due to their safety and biocompatibility. These carriers not only protect GABA from degradation in the harsh gastrointestinal environment but also improve its intestinal absorption through optimized particle size or surface modifications, thereby increasing its potential for transport across the BBB into the central nervous system (CNS) [57,58]. For example, encapsulating GABA within whey protein nanoparticles or chitosan microspheres can effectively shield it from enzymatic degradation and acidic conditions in the gut [59,60,61]. Furthermore, nanoparticles targeting receptors such as ferritin or the insulin receptor can undergo receptor-mediated transcytosis across the BBB, enabling central delivery of GABA [45].

In summary, the understanding of GABA transport across the BBB has evolved through three key stages: from the initial concept of an absolute barrier, to recognizing its dynamic regulatory nature, and subsequently exploring underlying transport mechanisms. The concurrent development of delivery technologies has significantly advanced our ability to overcome BBB limitations and improve the bioavailability of GABA in the CNS. These foundational studies not only deepen our understanding of GABAergic neurotransmission regulation but also provide a basis for developing new strategies to alleviate symptoms related to sleep disorders and anxiety. For instance, modulating the activity of specific transporters or altering BBB permeability could increase GABA concentration in the brain, offering targets for dietary GABA supplementation to improve sleep [21]. Nevertheless, several critical gaps remain in our understanding of GABA transport across the BBB. First, does dietary GABA differentially regulate the expression and functional activity of GATs on BBB endothelial cells under different physiological states? Second, given the dynamic nature of BBB structure and function, do its permeability and transporter activity fluctuate cyclically with the sleep–wake cycle, thereby influencing the efficiency of GABA entry into the brain? Third, are there significant differences in the distribution, subtype expression, and regulatory mechanisms of GATs and other potential transporters across different brain regions? Fourth, the clinical translation of current delivery technologies faces limitations: data on the intestinal absorption efficiency and long-term safety of oral nanocarriers are insufficient; and delivery systems optimized explicitly for GABA are lacking.

Addressing these questions is essential for a comprehensive understanding of the physiological and pharmacological mechanisms by which GABA crosses the BBB to regulate sleep. Accordingly, future research should focus on the following directions: First, utilizing combined animal models and human clinical trials to investigate the effects of different dietary patterns (e.g., single high-dose vs. chronic low-dose intake) on BBB transporters, and identifying key transport pathways for GABA under physiological conditions. Second, applying neuroimaging techniques (e.g., functional MRI, PET) coupled with circadian rhythm monitoring to analyze variations in BBB permeability and transporter activity across the sleep–wake cycle, informing optimal timing for GABA intervention. Third, employing single-cell sequencing and in situ hybridization to map the distribution of GABA transporters in sleep-regulating brain regions, elucidating region-specific transport mechanisms to provide targets for precise delivery. Forth, optimizing the design of food-grade delivery carriers, for example, through surface modification (e.g., with targeting ligands for the transferrin receptor) to enhance brain targeting, while conducting long-term human trials to validate their safety and efficacy, thereby promoting the industrial application of delivery technologies for GABA.

## 4. Mechanisms of GABA in Sleep Improvement via Metabolic Pathways

Beyond direct GABA_A_ receptor activation, recent research has highlighted the complex roles of GABA metabolites in regulating sleep–wake cycles, maintaining energy homeostasis, providing neuroprotection, and influencing multisystem interactions. While GABA is classically metabolized via GABA transaminase (GABA-T) into the tricarboxylic acid (TCA) cycle for energy production, its metabolic network is broader. For instance, succinic semialdehyde (SSA) can be converted by succinic semialdehyde reductase (SSR) into γ-hydroxybutyrate (GHB), establishing GHB as a key endogenous GABA metabolite. GABA metabolites can also indirectly influence sleep via inflammation and oxidative stress. This section covers GHB’s dual regulation, discoveries in GABA metabolism, and the interactions of metabolites with sleep–wake regulation.

### 4.1. Dual Regulatory Mechanisms of GHB

As a key endogenous metabolite of GABA, GHB exhibits a distinct mechanism for sleep improvement, characterized by dual regulation. It may serve as a bridge molecule through which orally supplemented GABA indirectly influences brain function [62]. GHB is intricately linked to GABA metabolism through the common intermediate (SSA), forming a bidirectional metabolic cycle that allows GHB to both replenish GABA reserves and participate independently in neuronal signaling [63].

Furthermore, GHB has a dual origin, derived from both endogenous GABA metabolism and exogenous precursors, underscoring its pivotal role in the neurotransmitter metabolic network [64].

Mechanistically, GHB primarily promotes sleep through the activation of GABA_B_ receptors. This action prolongs NREM sleep duration, enhances sleep depth, and reduces excessive daytime sleepiness, explaining its unique efficacy in treating narcolepsy [65,66]. However, GHB’s effects are not solely dependent on GABA_B_ receptor activation. Studies show that exogenous GHB supplementation can significantly increase total sleep time and reduce awakenings even under low sleep pressure [61]. Long-term GHB administration induces neuroadaptive changes within the GABAergic system, sustaining sleep-promoting effects [67]. Further mechanistic exploration revealed that GHB can act primarily on the suprachiasmatic nucleus (SCN, the master circadian clock) and the thalamic reticular nucleus (a key region for sleep rhythm integration),with the concentration of GHB in the brain typically exists at concentrations ranging from 0.1 to 1 µM, which can be influenced by factors such as age, health status, and physiological conditions [68]. By modulating neuronal firing rates in these areas, GHB optimizes the physiological rhythm of sleep architecture rather than merely inducing sedation [65]. Subsequent neuroimaging studies in humans have provided further evidence for GHB’s complex modulation of large-scale brain networks. For instance, fMRI studies in healthy volunteers have shown that GHB can enhance functional connectivity between key networks involved in salience detection and executive control, such as the anterior cingulate cortex (ACC) and the central executive network (CEN). This reorganization of network dynamics is thought to underpin the transition to a sleep-prone brain state and the improvement in sleep quality, without inducing excessive sedation [69].

Such mechanistic insights offer promising avenues for developing sleep-regulating functional foods, particularly those rich in GABA or GHB precursors. However, some risks associated with high-dose exogenous GHB-including respiratory depression, cognitive impairment, and abuse potential-warrant caution in its direct clinical application [66,67].

Although there is the dependence potential of GHB, current evidence suggests that GABA intake through food is generally safe, a conclusion supported by three key factors.

First, dosage is a critical determinant. The typical prescribed dose of pharmaceutical GHB ranges from several grams per administration, whereas the common daily intake of dietary GABA is typically between 100–300 mg. Then after metabolic conversion, results in plasma and brain GHB concentrations substantially lower than pharmacological doses, insufficient to activate the high-affinity binding sites associated with its dependence liability [70].

Secondly, an intrinsic metabolic buffering system exists. As previously mentioned, a tightly regulated, bidirectional metabolic cycle operates between GABA and GHB [64]. The introduction of GABA at physiological concentrations is processed within this network, maintaining GHB homeostasis and preventing its pathological accumulation [71].

Finally, multiple clinical trials on GABA supplements have reported no serious adverse effects related to GHB over intervention periods lasting several weeks. GABA holds a “Generally Recognized as Safe” (GRAS) status in the United States and is approved for use as a food additive in many countries [70].

Collectively, while understanding the metabolic link between GABA and GHB is mechanistically critical, the available data support the safety of GABA intake at conventional dietary and recommended doses. Several critical knowledge gaps persist in this field: the in vivo conversion efficiency of dietary GABA to GHB and the resulting physiological concentrations-decisive parameters for evaluating the efficacy and safety of GABA-based functional foods-remain uncharacterized; meanwhile, the influence of individual genetic variations (e.g., GHB receptor polymorphisms) on sensitivity to GHB and responses to GABA interventions remains unclear, directly hindering the development of precision nutrition strategies. To address these challenges, future research should focus on two key directions: first, exploring novel approaches to achieve low-dose, sustained endogenous GHB supply through GABA-enriched foods; and second, developing selective GHB receptor agonists that preserve physiological sleep-promoting benefits while minimizing risks mediated by GABA_B_ receptor activation.

### 4.2. New Discoveries in GABA Metabolic Pathways

Research on GABA metabolism has witnessed groundbreaking advances in recent years. The traditional view held that GABA is primarily metabolized by GABA-T into SSA, which is subsequently oxidized to succinate and enters the TCA cycle, ultimately contributing to ATP synthesis for neuronal energy supply. This process is particularly critical for maintaining energy metabolic homeostasis in the brain, as it rapidly replenishes energy consumed by neurons in regions with high neural activity demands, such as the hypothalamus and hippocampus [72]. While this classical pathway underscores GABA’s dual role as both a neurotransmitter and an energy substrate, recent studies have progressively revealed the complexity of its metabolic network, demonstrating that GABA metabolism is not a single linear pathway but rather a multi-branched, multifunctional regulatory system.

A pivotal recent discovery concerns the bidirectional conversion of SSA, representing a core branch point in GABA metabolism. Beyond the classical pathway where SSA is oxidized by succinic semialdehyde dehydrogenase (SSADH) to enter the TCA cycle, SSA can also be catalyzed explicitly by SSR in an NADPH-dependent reaction to produce GHB. The activity of this reaction is significantly higher in specific brain regions, such as the thalamic reticular nucleus and the suprachiasmatic nucleus of the hypothalamus, directly establishing GHB’s central role as a key endogenous metabolite of GABA [73]. More importantly, GHB can be reversibly converted back to SSA via GHB dehydrogenase, and SSA can then be transformed back to GABA by GABA-T, forming a closed-loop metabolic cycle between GABA and GHB. Such a metabolic cycle not only replenishes neurotransmitter reserves during periods of high GABA consumption (e.g., under stress) but also balances the intracerebral concentrations of GABA and GHB by regulating metabolic flux, thereby preventing neurotoxicity associated with an excess of either compound [74].

From a holistic metabolic perspective, GABA resides at the intersection of the neurotransmitter system and the broader metabolic network. The decarboxylation of glutamate to GABA, catalyzed by glutamate decarboxylase (GAD, including isoforms GAD65 and GAD67), is a vitamin B6-dependent process. While the constitutive expression of GAD67 maintains basal GABA levels, the inducible expression of GAD65 responds to surging GABA demands under stress. Concurrently, the degradation of GABA by GABA-T is coordinated with the action of glutamine synthetase (GS). GS utilizes the ammonium ions produced during GABA metabolism to convert glutamate into glutamine. Glutamine, in turn, can be hydrolyzed back to glutamate by glutaminase [70]. This transcellular metabolic collaboration, where neurons primarily synthesize GABA and astrocytes metabolize GABA while regenerating glutamate, not only maintains the balance between excitatory and inhibitory neurotransmission but also regulates metabolic coupling between glial cells and neurons. For instance, astrocytes can supply glutamate precursors to neurons via this cycle, ensuring the sustained synthesis of GABA.

Notably, various intermediates within the GABA metabolic network exhibit sleep-regulatory activities through diverse mechanisms. Beyond GHB’s dual-receptor modulation(via the GABA_B_ receptor and a specific GHB receptor), aberrant SSA metabolism can induce cerebral oxidative stress and inflammatory responses. For example, in SSADH-deficient mouse models, SSA accumulation leads to increased production of reactive oxygen species and upregulation of pro-inflammatory factors, such as IL-6, thereby disrupting the sleep–wake cycle. Furthermore, the link between GABA metabolism and the TCA cycle allows for indirect influence on sleep via energy metabolism. When the flux of GABA into the TCA cycle via the classical pathway increases, the duration of slow-wave sleep (SWS) in brain regions like the hippocampus and cortex is significantly prolonged. This effect may be related to the activation of sleep-regulatory neurons, such as those in the ventrolateral preoptic area(VLPO)by metabolic products like ATP. These findings collectively indicate that GABA metabolism is not merely a process of material conversion, but constitutes a functional network that interconnects energy homeostasis, neural signaling, and sleep regulation [75].

### 4.3. Association Between Metabolites and Sleep–Wake Regulation

The GABAergic system exhibits significant functional heterogeneity and mechanistic diversity in regulating sleep and wakefulness. Although GABA is traditionally thought to promote sleep primarily via GABA_A_ receptors [62,76], recent studies have revealed brain region-specific functions. In specific neural circuits, GABAergic transmission is conversely associated with the maintenance of wakefulness [62]. This bidirectional regulatory characteristic indicates that the impact of GABA on sleep and wakefulness is highly dependent on the specific neural circuitry involved. For instance, within the SCN, the master circadian clock, GABAergic neurons help maintain sleep rhythms by inhibiting the release of wake-promoting factors during the night [77]. In contrast, GABAergic transmission in the brainstem’s locus coeruleus may indirectly regulate the threshold for maintaining wakefulness by antagonizing the activity of noradrenergic neurons [62,78]. Such regional functional differences further highlight the complexity of GABAergic system regulation.

From an energy metabolism perspective, distinct metabolic differences exist between sleep and wake states. During wakefulness, the brain utilizes energy-efficient oxidative phosphorylation to conserve metabolites; during sleep, energy and metabolites are allocated to support protein synthesis and turnover [79]. GABA metabolism, being a key component of cerebral energy homeostasis, likely undergoes dynamic changes that are “coupled” to the energy demands of the sleep–wake cycle. During NREM sleep, the efficiency of GABA entering the tricarboxylic acid (TCA) cycle via the “GABA shunt” is significantly enhanced, potentially providing additional ATP for neuronal repair. Concurrently, the accumulation of the GABA metabolic intermediate succinic semialdehyde (SSA) may indirectly reduce neuronal excitability by inhibiting mitochondrial respiratory chain complex activity, providing a molecular basis for energy “conservation” during sleep [80,81,82]. The interplay between metabolic regulation and neural activity jointly contributes to the stability of the sleep–wake cycle, and its dysregulation may be a significant factor in sleep disorders associated with disturbances in cerebral energy metabolism, such as insomnia [73,83]. The GABAergic system also interacts extensively with other sleep-regulatory pathways, often relying on metabolites as signaling intermediaries. Melatonin, a primary hormone that regulates the sleep–wake cycle, communicates with the master clock in the SCN and with peripheral clocks in other cells and organs. Research has shown that the GABA metabolite γ-hydroxybutyrate (GHB) can enhance melatonin’s regulatory effect on the circadian clock by increasing the expression of melatonin receptors (MT1/MT2) in the SCN region. During the peak nighttime sleep period, brain GHB concentrations correlate positively with melatonin levels, and exogenous GHB supplementation can prolong melatonin-induced NREM sleep by 15–20% [60]. These findings suggest that GABA metabolites function as synergistic modulators for the melatonin pathway, reinforcing sleep rhythm regulation. Furthermore, in mouse models of Alzheimer’s disease (AD), researchers observed abnormalities in sleep-related neuronal activity. They decreased sleep quality, accompanied by reduced activity of GABA metabolic enzymes (e.g., GABA-T) and overactivation of the melanin-concentrating hormone (MCH) system. Further investigation revealed that GABAergic neurons can inhibit MCH neuron firing by releasing GABA. In AD models, impaired inhibitory effects due to disrupted GABA metabolism lead to MCH-mediated hyperarousal, indicating that the interaction between GABAergic and MCH neurons may not only rely on direct neural connections but also occur through competitive binding of GABA metabolites (e.g., GHB) to MCH receptors [84]. These findings underscore the central role of the GABAergic network in integrating multisystem sleep regulation.

In terms of therapeutic applications, modulating GABAergic transmission remains a primary strategy for treating sleep disorders. Although benzodiazepines are effective [62], their action is primarily through non-specific activation of GABA_A_ receptors, leading to side effects like dependence and cognitive impairment, driving research into more precise interventions targeting GABA metabolic pathways. Studies have shown a synergistic effect between GABA and L-theanine in improving sleep, with their mixture significantly shortening sleep latency and increasing NREM sleep duration [76]. Regulating key enzymes in GABA metabolism has emerged as a new direction. Inhibiting GABA-T activity reduces GABA breakdown and concurrently slows the production rate of its metabolite, GHB. This “bidirectional regulation” avoids excessive neural inhibition from GABA overload while preventing sleep architecture disruption caused by GHB accumulation. In animal experiments, the combined use of a GABA-T inhibitor and low-dose GABA increased NREM sleep duration in mice by 22% without the sleep fragmentation commonly associated with traditional sedatives [58].

GABA derived from fermented foods and its associated metabolites have become a focus for functional food development due to their safety profile and multi-targeted, precise regulatory potential [85]. For example, pea protein hydrolysate fermented with *Lbp. plantarum* SYLB 0016 exhibits increased GABA content and demonstrates sleep-promoting activity in mice, characterized by increased sleep duration, shortened sleep latency, and improved sleep structure [86]. Furthermore, in *Aspergillus oryzae*-fermented germinated brown rice, an 8–10 fold increase in GABA content is accompanied by the production of the metabolic derivative γ-aminobutyric acid methyl ester (GABA-Me) [73]. GABA-Me may inhibit the activity of GHB dehydrogenase in the brain, thereby delaying the reverse conversion of GHB back to GABA and effectively prolonging the window for GHB’s sleep-promoting activity. Simultaneously, short-chain fatty acids (SCFAs) such as butyrate, generated during the fermentation process, can activate the intestinal GPR41 receptor. This activation indirectly upregulates the expression of the GABA-synthesizing enzyme GAD67 in the brain, establishing a collaborative metabolic regulatory axis between the gut and the central nervous system [28]. These findings suggest that specific fermented food matrices harbour multi-component synergistic networks, comprising GABA, metabolic enzymes, and gut-derived signalling molecules. The regulatory efficacy of these complex networks appears significantly superior to that of isolated GABA supplementation.

Of particular importance is the coupled balance between GABA metabolism and the glutamate-glutamine cycle, which is crucial for maintaining sleep homeostasis. Chronic stress can lead to glutamate accumulation in the brain, enhancing neuronal excitability via NMDA receptor activation while simultaneously suppressing the activity of GAD65/67 enzymes. This disruption skews the critical balance between excitatory (glutamate) and inhibitory (GABA) neurotransmission. Conversely, the GABA metabolic intermediate glutamine, shuttled from astrocytes to neurons, serves as a precursor for glutamate synthesis, forming an essential “metabolic buffer” mechanism. Clinical evidence demonstrates that supplementation with a combination of GABA and glutamine in insomnia patients reduced Pittsburgh Sleep Quality Index (PSQI) scores by 34% and restored the GABA/glutamate ratio in cerebrospinal fluid to normal levels [76]. These findings collectively suggest that modulating the excitatory–inhibitory neurotransmitter balance at the metabolic level represents a promising target for precision interventions in sleep disorders. Future research should prioritize elucidating the activity patterns of key GABA metabolic enzymes across different sleep stages (e.g., rapid eye movement(REM) vs. NREM). It is also critical to define the regulatory specificity of metabolites on neural circuits within distinct brain regions. Integrating these findings with population-based metabolomics data will be instrumental in developing personalized GABA intervention strategies tailored to individual metabolic profiles [87].

## 5. Application and Future Research Directions

### 5.1. Summary

**Figure 2:**
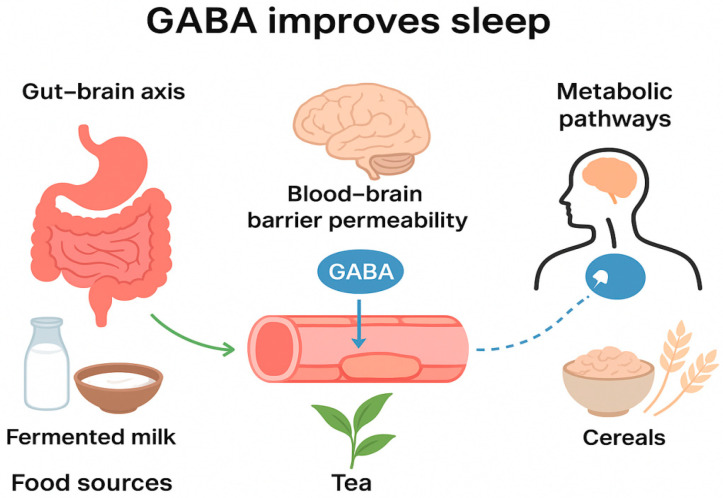
Core mechanisms of dietary GABA (sourced from fermented milk, grains, tea, etc.) in improving sleep via the gut–brain axis, BBB, and metabolic pathways. The gut–brain axis comprises the vagus nerve, the HPA axis, and the immune pathways. The BBB pathway involves dynamic regulation of tight junctions, transporters, and delivery innovations. The metabolic pathway centers on the GABA-GHB cycle, linking energy homeostasis and multi-receptor regulation. The three pathways interconnect synergistically.

This review synthesizes evidence establishing a multi-dimensional and synergistic regulatory network through which dietary GABA improves sleep, moving beyond the traditional view of GABA as solely a central inhibitory neurotransmitter. As illustrated in Figure 2, GABA from specific food sources (e.g., fermented dairy, tea, fermented grains, and legumes) exerts a multi-dimensional, cross-system regulatory influence on sleep improvement via three primary pathways: the gut–brain axis, targeted penetration of the BBB, and the regulation of endogenous metabolic networks.

We have elaborated on three core pathways—the gut–brain axis, the BBB, and endogenous metabolic networks—not as independent routes but as interconnected components of an integrated “gut–brain-metabolism” model.

The gut–brain axis serves as a critical peripheral foundation. GABA can influence central sleep–wake circuits via vagal afferent signaling, HPA axis regulation, and immune–inflammatory modulation. This axis provides a fundamental communication layer that sets the stage for central effects. The traditional perception of the BBB as an absolute barrier has been revised currently. The dynamic regulation of tight junctions, the involvement of specific transporters (e.g., GATs), and receptor-mediated transcytosis collectively determine the brain’s accessibility to peripheral GABA. This understanding paves the way for innovative brain-targeted delivery technologies, such as nanocarriers and focused ultrasound, to enhance GABA’s central bioavailability.

Furthermore, the role of GABA extends profoundly into cerebral metabolic regulation. The metabolic network centered around the “GABA shunt” and the GABA-GHB cycle effectively couples neuronal energy homeostasis with sleep–wake control. Key metabolites like GHB exert “physiological sleep-mimicking” effects through multi-receptor targeting (GABA and specific GHB receptors), while intermediates such as glutamine help maintain the crucial excitatory–inhibitory neurotransmitter balance.

Critically, these three pathways do not operate in isolation. They engage in continuous crosstalk: gut–brain axis signaling can influence BBB permeability and central metabolic states; dynamic BBB transport dictates the intracerebral levels of GABA and GHB; and the central metabolic network can, in turn, provide feedback that modulates gut function and overall signaling efficiency. This synergistic interplay forms a complete, self-regulating system that underlies GABA’s holistic and physiological sleep-promoting effects.

In conclusion, the paradigm for GABA-mediated sleep improvement is shifting from a focus on isolated neurotransmitter modulation to an integrated, system-level understanding. This comprehensive framework not only deepens our mechanistic knowledge but also provides a robust theoretical foundation for innovating the next generation of functional foods, targeted delivery systems, and personalized nutritional strategies for sleep health management.

### 5.2. Application

The theoretical foundation of GABA’s multi-mechanistic role in sleep improvement via the gut–brain axis, the blood–brain barrier, and metabolic pathways provides a basis for its potential translational applications. Current research and development have begun to establish a framework encompassing upstream raw material production, midstream clinical validation, and downstream product development.

At the level of raw material preparation, microbial fermentation represents one of the effective strategies for GABA enrichment. As shown in Table 1, the use of specific strains (e.g., *Levilactobacillus brevis* and *Lbp. plantarum*) and optimized fermentation conditions can significantly increase GABA content in matrices such as pea protein hydrolysate, dairy products, and germinated brown rice, providing potential raw material sources for related functional foods. Future efforts could further optimize fermentation processes by considering the characteristics of different food matrices and the potential synergistic effects between GABA and other active ingredients.

In terms of clinical validation, existing studies have accumulated evidence supporting the role of GABA in sleep improvement. Table 2 summarizes clinical trials indicating that GABA interventions-whether as a single component or in combination with ingredients such as L-theanine-show positive trends in reducing sleep latency and improving sleep quality, generally with good tolerability. These results provide preliminary clinical support for GABA’s application. Furthermore, clinical studies have confirmed that GABA-based interventions can positively impact sleep. For instance, the combined use of GABA (100 mg) and Apocynum venetum leaf extract (50 mg) synergistically shortened sleep latency and increased NREM sleep time, with effects significantly superior to either component alone. Additionally, intervention with GABA-producing Bifidobacterium breve BLa80 for 4 weeks reduced sleep onset time in healthy adults and modulated the gut microbiota ratio of Bacteroidetes to Firmicutes. Given the complex bidirectional relationship between sleep disorders and anxiety-where sleep deprivation exacerbates anxiety symptoms, which in turn impair sleep quality-enhancing GABAergic activity is regarded as a key strategy for dual improvement. Future multicenter, large-sample clinical studies are warranted to validate the effects of GABA combination regimens (e.g., GABA with apigenin) in populations with comorbid sleep disorders and anxiety, and to determine optimal dosages and intervention durations.

At the level of market translation, based on the above research foundation, a variety of dietary supplements and functional foods featuring GABA as a primary component have emerged in the market. As shown in Supplementary Table S3, these products often incorporate GABA in combination with other ingredients (e.g., L-theanine, herbal extracts) to meet diverse health needs, reflecting an exploratory trend in translating scientific research into practical applications. Looking ahead, further optimization of food-grade delivery systems-such as improving the targeting precision and oral bioavailability of nanocarriers-coupled with long-term human trials to validate their safety, will be essential for advancing the industrial application of GABA-based interventions.

### 5.3. Future Research Directions

Future research could strive for breakthroughs in the mechanistic depth of mechanism, technological innovation, and clinical translation under this ”gut–brain-metobolism” regulatory network. To bridge the gap from theory to application, the following directions are particularly critical and urgent:

1. Dynamic and Precise Mechanistic Investigations. Future efforts can focus on elucidating the dynamic processes of GABA-mediated sleep regulation as current studies are largely static and descriptive. This includes integrating multi-omics technologies with neuroimaging techniques (e.g., fMRI, PET) to uncover the dynamic changes in GABA across the gut–brain axis, blood–brain barrier, and metabolic networks during different sleep–wake stages (e.g., NREM vs. REM); and employing single-cell sequencing to meticulously map the distribution of GABA receptors, transporters, and metabolic enzymes in key sleep-regulating brain regions (e.g., the suprachiasmatic nucleus, amygdala, thalamic reticular nucleus), thereby clarifying region-specific regulatory mechanisms. It is recommended to do some work to answer the few specific and testable research questions such as: Does the transport efficiency of GABA across the BBB and its subsequent metabolic flux in the brain undergo cyclical fluctuations in accordance with circadian rhythms and sleep–wake states? How specific probiotics (e.g., high-GABA-yielding *lactobacilli* strains) modulate the overall structure of the gut microbiota to indirectly enhance GABAergic signaling along the gut–brain axis?
2. Rational Design and Safety Validation of Next-Generation Probiotics for Food Applications and Fermentation Processes. Regarding strain and process optimization, future work should concentrate on employing synthetic biology to construct next-generation probiotics (NGPs) with high GABA yields, for instance, by increasing the copy number of glutamate decarboxylase (GAD) genes or utilizing gut-specific inducible promoters. It is a prerequisite for the application translation to systematically evaluate the intestinal colonization stability, immunogenicity, and long-term consumption safety of these engineered strains.
3. Standardization and Personalization of Clinical Research. To advance the precise application of GABA-based functional foods or drugs, there is an urgent need for multicenter, large-sample randomized controlled trials employing standardized sleep assessment metrics (e.g., polysomnography). Research should integrate population genomics, metabolomics, and microbiomics data to deeply analyze the impact of individual differences (e.g., GABA receptor gene polymorphisms, baseline gut microbiota composition) on intervention efficacy, thereby providing a basis for developing personalized nutritional strategies for sleep. For example, define the synergistic effects of GABA in combination with natural active compounds (e.g., apigenin, L-theanine) and establish optimal dosages and intervention durations for specific populations (e.g., insomnia patients with comorbid anxiety).
4. Future research should integrate pharmacokinetic methods to systematically elucidate the relationship between the plasma concentration-time profiles of different oral GABA doses (e.g., 50, 100, 300 mg) and corresponding sleep improvement outcomes (such as PSQI scores and polysomnographic parameters). This is crucial for determining the minimum effective dose and the optimal dosage range for GABA-based functional foods, ultimately bridging the gap between mechanistic understanding and practical application.
5. Innovation in Food-Grade Delivery Technologies and Industrialization of Functional Foods. To improve the central bioavailability of GABA, further optimization of food-grade nano-delivery systems (e.g., whey protein nanoparticles and chitosan microspheres) is necessary, potentially through surface modification with targeting ligands (e.g., transferrin receptor antibodies) to enhance brain targeting efficiency. Simultaneously, systematic in vitro and in vivo experiments, coupled with long-term human studies, are mandatory to evaluate the intestinal absorption efficiency, biosafety, and long-term consumption risks of these nanocarriers, thereby clearing obstacles for their industrialization.

## 6. Methods

This review was conducted in accordance with the PRISMA guidelines. Given the narrative and mechanistic focus of this review, which aimed to synthesize evidence from diverse study designs(including in vitro, animal, and human studies) rather than to quantitatively compare intervention effects, a meta-analysis was not performed. Nevertheless, a systematic search and screening process was rigorously followed to ensure comprehensiveness and reproducibility. We systematically searched multiple databases, including PubMed, Web of Science, and the China National Knowledge Infrastructure (CNKI). The search timeframe spanned from the inception of each database to 2025. The keywords covered three main themes: (1) GABA-related terms: “γ-aminobutyric acid (GABA)”, “GABA”; (2) Sleep-related terms: “sleep regulation”, “sleep improvement”, “insomnia”; and (3) Mechanism-related terms: “gut–brain axis”, “vagus nerve”, “hypothalamic–pituitary–adrenal axis (HPA axis)”, “immune pathway”, “blood–brain barrier (BBB)”, “BBB permeability”, “GABA transporters (GATs)”, “γ-hydroxybutyrate (GHB)”, “GABA metabolism pathway”, “GABA receptors”, “probiotics”, “next-generation probiotics (NGPs)”, “fermented food”, “functional food”, “nanocarrier”, “clinical trial”, “animal model”.

The search strategy was designed to retrieve records that contained at least one term from theme (1) AND at least one term from theme (2) AND at least one term from theme (3), using the Boolean operators “AND” to combine themes and “OR” to combine terms within each theme. We included the following types of publications: clinical trials, randomized controlled trials, narrative reviews, systematic reviews, and meta-analyses. The initial search yielded 2115 articles. After screening the titles and abstracts, 2010 articles that did not meet the eligibility criteria were excluded. The full texts of the remaining 105 articles were assessed, Ultimately resulting in the inclusion of 12 clinical studies (including randomized controlled trials, RCTs) focusing on sleep outcomes of GABA (e.g., intervention with GABA-enriched fermented milk, GABA-probiotic *Lbp. plantarum* Lp815, and GABA-complex formulations), 15 animal studies (covering murine, Drosophila, and rat models to evaluate sleep latency, NREM/REM sleep duration, and sleep structure), and 9 mechanistic studies (exploring the regulatory mechanisms of GABA on sleep via gut–brain axis, blood–brain barrier, and metabolic pathways) for the core analysis. Additional narrative reviews, systematic reviews, and meta-analyses that met the eligibility criteria were used to supplement mechanistic discussions and background context (the exact number was not quantified).

**Figure 3:**
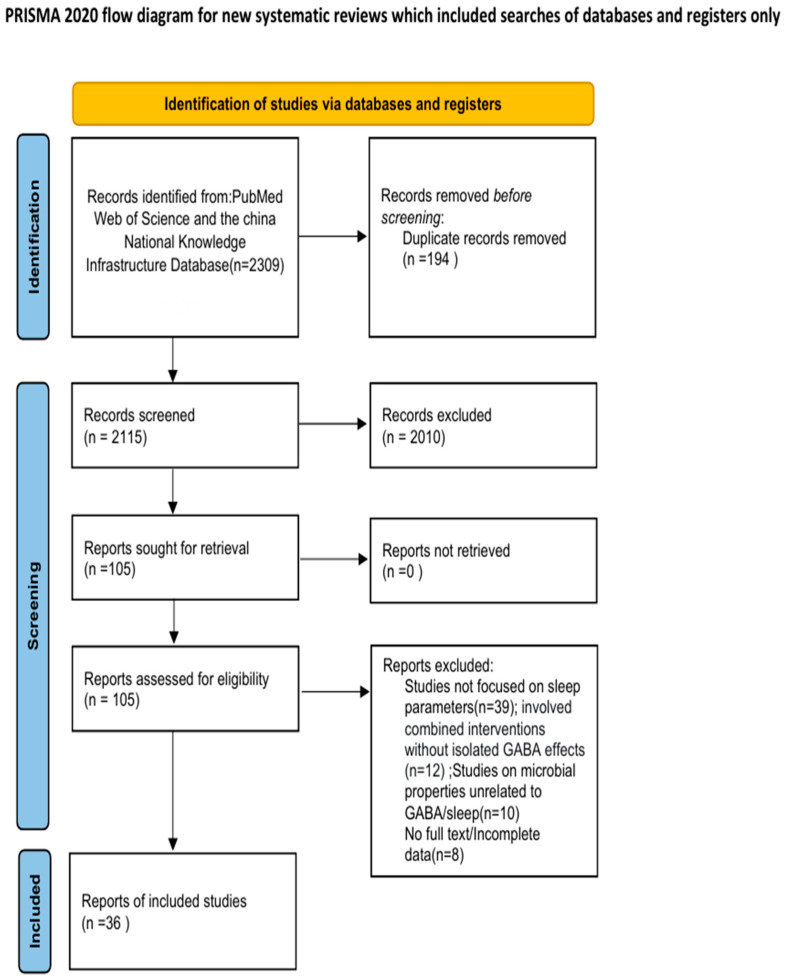
PRISMA flow diagram illustrating the study selection process. The diagram outlines the identification, screening, eligibility, and inclusion phases of the review. A total of 2115 records were identified through database searching. After the removal of duplicates, 2115 records were screened based on titles and abstracts, resulting in the exclusion of 2010 irrelevant studies. The full texts of the remaining 105 articles were assessed for eligibility. Studies were excluded for the following reasons: (a) studies not focused on sleep parameters (n = 39), (b) involved combined interventions without isolated GABA effects (n = 12), (c) studies on microbial properties unrelated to GABA/sleep (n = 10), or (d) no full text/Incomplete data (n = 8). Ultimately, 36 studies (12 clinical, 15 animal, and 9 mechanistic) were included in the qualitative synthesis. Additional narrative and systematic reviews were consulted for background and mechanistic context but are not represented in this flowchart.

The inclusion criteria were as follows: (1) studies investigating the effects or mechanisms of GABA on sleep improvement; (2) studies published in English or Chinese; and (3) for clinical or animal studies, the provision of at least one complete dataset of the following: (i) means ± standard deviations of sleep parameters (e.g., sleep latency, NREM duration) pre- and post-intervention, (ii) comparative differences in sleep indicators between intervention and control groups, or (iii) inclusion of a positive control group with statistical difference analysis. The exclusion criteria were: (1) studies reporting only GABA intervention outcomes without including sleep-related parameters; (2) studies reporting interventions that do not contain GABA or where the effect of GABA cannot be isolated (3) studies on microbial properties without discussing their relevance to sleep regulation by GABA. The flowchart of the literature screening process is presented in Figure 3.

Although a meta-analysis was not conducted in this review, we acknowledge that publication bias could potentially influence the conclusions. To address this concern, we implemented the following strategies: (1) We systematically searched multiple databases (including both English and Chinese databases) to cover as much of the published and grey literature as possible; (2) We performed backward and forward reference tracking of included articles to identify additional relevant studies that might have been missed; (3) We did not exclude studies based on their results (e.g., non-significant or negative findings) as long as they met the inclusion criteria. Given the limited number and high heterogeneity of the included studies, a funnel plot was not constructed for quantitative assessment. However, we believe that the included studies collectively provide a comprehensive overview of the mechanisms and effects of GABA in sleep improvement.

We analyzed the included studies. Given the broad scope of probiotic research, the search strategy was designed to be flexible, which may have introduced certain limitations. To address this, we supplemented the search by tracking references from key articles to ensure the inclusion of additional relevant studies. We conducted a risk-of-bias assessment for the included clinical trials and animal studies. For preclinical studies, the Cochrane Risk-of-Bias Tool (RoB 2.0) was used to evaluate RCTs, covering five domains: randomization process, deviations from intended interventions, missing outcome data, measurement of the outcome, and selective reporting. The assessment results are presented in Supplementary Table S1. For animal studies, the Systematic Review Centre for Laboratory Animal Experimentation (SYRCLE) risk-of-bias tool was applied, covering selection bias, performance bias, detection bias, attrition bias, reporting bias, and other biases. The evaluation results are shown in Supplementary Table S2. For clinical trials, the main limitation was unclear reporting of randomization, though outcome data and reporting biases were generally low-risk. For animal studies, key methodological details like blinding and randomization were often unreported. While these limitations warrant caution, the consistent sleep-improving effects observed across studies strengthen the reviewed conclusions.

## Figures and Tables

**Table 1:** Examples of GABA Enrichment in Different Food Matrices.

Food Matrix	Strain/Method Used	Optimal Parameters	GABA Yield/Enrichment Effect	Reference
Pea Protein Hydrolysate	*Lvl. brevis* SYLB 0016	Substrate 5%, pH 5.0, 37 °C, 48 h	~3.5 g/kg	[88]
Dairy Product	*Lvl.brevis* 877 G & Latilactobacillus *sakei* 795 co-fermentation	Skim milk, 29.57 mM MSG added	22.51 mM	[89]
Germinated Brown Rice	Water soaking (endogenous GAD activation)	40 °C, pH 4.0, with an 8-h immersion procedure.	Increase 8–10 fold (~300 mg/100 g) vs. non-germinated	[90]
Kimchi	*Lbp. plantarum* K255	MRS broth, 2% MSG added	163.6 mg/mL	[91]
Kimchi	*Leuconostoc citreum* S5 & L. *plantarum* KS2020 co-fermentation	Plant-based medium, 5% sucrose	Viable count reached 9.42 log CFU/mL	[92]

**Table 2:** Selected Clinical Studies on GABA Intervention for Sleep Improvement.

Active Component	Study Design	Basic Research Parameters	Dose/Intervention	Main Outcomes	Reference
GABA & Apocynum venetum Leaf Extract (AVLE)	EEG measurement	PSQI ≥ 6; Sample size: 16 subjects; Study duration: 2 weeks	GABA (100 mg) and/or AVLE (50 mg)	GABA: reduced sleep latency 5.3 min; AVLE: increased NREM sleep time 7.6%; Combination: synergistic effects	[93]
GABA & L-Theanine	Animal study	8-week-old male mice; Study duration: 9 days	GABA and L-theanine mixture	Reduced sleep latency, improved NREM sleep.	[76]
Oral GABA	Randomized, single-blind, placebo-controlled, crossover	PSQI ≥ 6; Sample size: 10 subjects; Study duration: 2 weeks	Single oral GABA dose	Significantly shortened sleep latency, increased total NREM sleep time.	[94]
Probiotic *Lp815 Lbp. plantarum*	Randomized, double-blind, placebo-controlled	Self-reported individuals with sleep disorders; Sample size: 139 subjects; Study duration: 6 weeks	*L. plantarum* Lp815 (GABA-probiotic)	Improved sleep, reduced anxiety, and increased urinary GABA.	[5]
GABA & Asparagine Powder	Group trial	PSQI ≥ 6; Sample size: 54 subjects; Study duration: 2 weeks	Group A: GABA 120 mg + Asparagine 1500 mg; B: GABA 120 mg; C: Placebo	GABA and asparagine powder are beneficial for sleep (as assessed by the PSQI).	[95]
Poria Cocos, Ziziphus spinose, & GABA Combination	Randomized, double-blind, controlled trial	PSQI ≥ 7;Sample size: 70 subjects; Study duration: 4 weeks	Combination intervention for 4 weeks	Improved sleep quality, increased skin hydration, reduced skin roughness	[96]
*Bifidobacterium animalis* subsp. *lactis* BLa80 (GABA-producing in vitro)	Randomized, placebo-controlled	6 ≤ PSQI ≤ 18; Sample size:106 subjects; Study duration: 8 weeks	*B. lactis* BLa80	Improving sleep quality in healthy individuals and regulating gut microbiota.	[97]

## Data Availability

No new data were created or analyzed in this study. Data sharing is not applicable to this article.

## Supplementary Materials

The following supporting information can be downloaded at https://www.mdpi.com/article/10.3390/foods14223856/s1. Table S1: Risk-of-bias summary judgments about each risk-of-bias item for each included clinical study; Table S2: Risk-of-bias summary judgements about each risk-of-bias item for each included animal study; Table S3: Overview of selected marketed dietary supplements and functional foods containing GABA for sleep support.
