# Supplementary material for: Progress in Research on the Mechanism of GABA in Improving Sleep
Source: Foods. 2025 Nov 11;14(22):3856. doi: 10.3390/foods14223856 (PMC12651798; doi:10.3390/foods14223856)
Supplement: Supplementary file 1 [file foods-14-03856-s001.zip › risk-of-bias assessment.pdf]

Supplementary Table S1. Risk of bias summary Judgements about each risk of bias item for each included clinical study.

Study	Year	Randomization	Missing Data	Outcome Measurement	Selective Reporting	Other Bias
Yamatsu et al.	2015	unclear	low risk	low risk	low risk	unclear
Kim et al.	2019	unclear	low risk	low risk	low risk	unclear
Yamatsu et al.	2016	unclear	low risk	unclear	low risk	unclear
GABA Probiotic Lp815 Study	2023	low risk	low risk	low risk	low risk	low risk
GABA + Asparagus Study	2022	unclear	low risk	low risk	unclear	unclear
Poria + Ziziphus + GABA Study	2024	unclear	low risk	low risk	low risk	unclear
B. lactis BLa80 Study	2025	low risk	low risk	low risk	low risk	low risk
GABA + AVLE Study	2015	unclear	low risk	low risk	low risk	unclear

Study	Year	Randomization	Missing Data	Outcome Measurement	Selective Reporting	Other Bias
GABA + L-theanine Study	2019	unclear	unclear	low risk	low risk	unclear
Oral GABA Crossover Trial	2016	unclear	low risk	unclear	low risk	unclear
GABA + Nanocarrier Trial	2024	unclear	unclear	unclear	unclear	unclear
GABA + Behavior Therapy Trial	2025	unclear	unclear	low risk	unclear	unclear

Supplementary Table S2. Risk of bias summary Judgements about each risk of bias item for each included animal study.

Study	Year	Random Sequence	Baseline	Allocation Concealment	Blinding	Random Housing	Outcome Assessment	Attrition	Selective Reporting	Other Bias
Park et al.	2017	unclear	low risk	unclear	unclear	unclear	unclear	low risk	unclear	low risk
Sun et al.	2020	unclear	unclear	unclear	unclear	unclear	unclear	low risk	unclear	low risk
Liu et al.	2017	unclear	unclear	unclear	unclear	unclear	unclear	low risk	unclear	low risk
Sun et al.	2023	unclear	unclear	unclear	unclear	unclear	unclear	low risk	unclear	low risk
Lee et al.	2024	unclear	unclear	unclear	low risk	unclear	unclear	low risk	unclear	high risk
Li et al.	2022	unclear	unclear	unclear	low risk	unclear	unclear	unclear	unclear	low risk
Huang et al.	2018	unclear	unclear	unclear	low risk	unclear	unclear	unclear	unclear	high risk
Zhang et al.	2022	unclear	low risk	unclear	unclear	unclear	unclear	low risk	unclear	unclear
Kim et al. (pea protein)	2025	unclear	low risk	unclear	unclear	unclear	unclear	low risk	unclear	low risk
GABA + FUS Study	2024	unclear	unclear	unclear	unclear	unclear	unclear	unclear	unclear	unclear

Study	Year	Random Sequence	Baseline	Allocation Concealment	Blinding	Random Housing	Outcome Assessment	Attrition	Selective Reporting	Other Bias
GABA + Chitosan Study	2024	unclear	unclear	unclear	unclear	unclear	unclear	unclear	unclear	unclear
GABA + Lactobacillus brevis	2023	unclear	low risk	unclear	unclear	unclear	unclear	low risk	unclear	low risk
GABA + Brown Rice Study	2022	unclear	unclear	unclear	unclear	unclear	unclear	unclear	unclear	unclear
GABA + Kimchi Study	2025	unclear	unclear	unclear	unclear	unclear	unclear	low risk	unclear	low risk
GABA + Tea Study	2023	unclear	unclear	unclear	unclear	unclear	unclear	low risk	unclear	unclear
