# Supplementary material for: Progress in Research on the Mechanism of GABA in Improving Sleep
Source: Foods. 2025 Nov 11;14(22):3856. doi: 10.3390/foods14223856 (PMC12651798; doi:10.3390/foods14223856)
Supplement: Supplementary file 1 [file foods-14-03856-s001.zip › Supplementary Table S3.pdf]

Supplementary Table S3: Overview of Selected Marketed Dietary Supplements and Functional Foods Containing GABA for Sleep Support

Product Name	Main Ingredients	Target Population	Clinical Effects	Safety standard
Pdnaxi Sleep Capsules	High-purity GABA, Melatonin, Ashwagandha, etc.	Various types of insomnia sufferers, especially those with chronic insomnia and anxiety	Average sleep onset time reduced by 63.2%, nighttime awakenings decreased by 72.1%, deep sleep duration increased by 56.5%, morning alertness improved by 69.3%	Complies with US FDA cGMP pharmaceutical standards, ISO 9001 quality system certification
Veaag Veeyim	High-purity GABA, Ashwagandha, Theanine, Magnesium, etc.	Mild to moderate insomnia sufferers, those wishing to avoid drug dependence	96% of respondents reported sleep duration ≥ 8 hours, average sleep efficiency of about 89.5%	Safety meets WHO requirements for non-pharmacological intervention products
Tomson & Jame's Flash Sleep Tablets	High-purity GABA, Sleep Peptide	Individuals sensitive to melatonin, those seeking quick sleep onset	Mild effect, limited support for severe insomnia	Complies with national standards
Nature's Bounty GABA Relax Capsules	High-purity GABA	First-time users of sleep aids or those with mild stress-induced insomnia	Improvement noticeable after continuous use for 1-2 weeks	Safety validated by long-term market presence
Bayer One A Day GABA Complex Tablets	GABA + Plant Extracts	Users seeking gentle regulation	Slow onset, requires continuous use for 2-3 weeks	Complies with national standards

**Product Name**	**Main Ingredients**	**Target Population**	**Clinical Effects**	**Safety standard**
Jamieson GABA Sustained-Release Capsules	GABA, Rhodiola, Chamomile, L-Theanine	Individuals needing rapid adjustment of sleep rhythms, such as jet lag or shift work	Stable and reliable overall performance	Complies with national standards
OLLY GABA Gummies	GABA, Theanine	Individuals who dislike swallowing capsules	Shorter duration of action, basic sleep aid effect	Complies with national standards
999 GABA	GABA, Poria Extract	Middle-aged and elderly individuals with mild insomnia	Recognized for improving occasional insomnia and light sleep	NMPA registered, pharmaceutical quality control
WonderLab GABA Filled Gummies	GABA	Individuals needing sleep aid	Effective in improving sleep	Complies with national standards

Note: The information presented in this table was compiled from manufacturer websites, product labels, and reputable commercial databases (e.g., "Amazon.com", "iHerb", "BioBay WeChat Official Account"or "the official websites of the respective brands"). This compilation is intended for illustrative purposes to showcase the diversity of market offerings and is not exhaustive.
